# Prognostic Impact of the Get-with-the-Guidelines Heart-Failure Risk Score (GWTG-HF) after Transcatheter Aortic Valve Replacement in Patients with Low-Flow–Low-Gradient Aortic Valve Stenosis

**DOI:** 10.3390/diagnostics13071357

**Published:** 2023-04-06

**Authors:** Clemens Eckel, Johannes Blumenstein, Oliver Husser, Dagmar Sötemann, Christina Grothusen, Judith Schlüter, Marc Becher, Holger Nef, Albrecht Elsässer, Georg Nickenig, Helge Möllmann, Vedat Tiyerili

**Affiliations:** 1 Department of Cardiology, St.-Johannes-Hospital, 44137 Dortmund, Germany; 2 Department of Cardiology, University of Oldenburg, 26129 Oldenburg, Germany; 3 Department of Cardiovascular Surgery, University of Schleswig Holstein, Campus Kiel, 24118 Kiel, Germany; 4 Department of Cardiology, University of Bonn, Heart Center Bonn, 53113 Bonn, Germany; 5 Department of Cardiology, University of Gießen, 35390 Gießen, Germany

**Keywords:** heart failure, THV, low flow–low gradient, aortic valve stenosis, get-with-the-guidelines heart-failure risk score, GWTG-HF, mortality, hospitalization for heart failure, TAVI

## Abstract

Objectives: This study examined the prognostic value of the get-with-the-guidelines heart-failure risk score (GWTG-HF) on mortality in patients with low-flow–low-gradient aortic valve stenosis (LFLG-AS) after transcatheter aortic valve implantation (TAVI). Background: Data on feasibility of TAVI and mortality prediction in the LFLG-AS population are scarce. Clinical risk assessment in this particular population is difficult, and a score has not yet been established for this purpose. Methods: A total of 212 heart failure (HF) patients with real LFLG-AS were enrolled. Patients were classified into low-risk (*n* = 108), intermediate-risk (*n* = 90) and high-risk (*n* = 14) groups calculated by the GWTG-HF score. Clinical outcomes of cardiovascular events according to Valve Academic Research Consortium (VARC-2) recommendations and composite endpoint of death and hospitalization for heart failure (HHF) were assessed at discharge and 1 year of follow-up. Results: Baseline parameters of the groups showed a median age of 81.0 years [77.0; 84.0] (79.0 vs. 82.0 vs. 86.0, respectively *p* < 0.001), median EuroSCORE II of 6.6 [4.3; 10.7] (5.5 vs. 7.2 vs. 9.1, *p* = 0.004) and median indexed stroke volume of 26.7 mL/m^2^ [22.0; 31.0] (28.2 vs. 25.8 vs. 25.0, *p* = 0.004). The groups significantly differed at follow-up in terms of all-cause mortality (10.2 vs. 21.1 vs. 28.6%; *p* < 0.035). There was no difference in intrahospital event rate (VARC). Postprocedural mean gradients were lower in high-risk group (7.0 vs. 7.0 vs. 5.0 mmHg, *p* = 0.011). No differences in postprocedural aortic valve area (1.9 vs. 1.7 vs. 1.9 cm^2^, *p* = 0.518) or rate of device failure (5.6 vs. 6.8 vs. 7.7%, *p* = 0.731) could be observed. After adjustment for known predictors, the GWTG score (HR 1.07 [1.01–1.14], *p* = 0.030) as well as pacemaker implantation (HR 3.97 [1.34–11.75], *p* = 0.013) turned out to be possible predictors for mortality. An increase in stroke volume index (SVI) was, in contrast, protective (HR 0.90 [0.83–0.97]; *p* = 0.006). Conclusions: The GWTG score may predict mortality after TAVI in LFLG-AS HF patients. Interestingly, all groups showed similar intrahospital event and mortality rates, independent of calculated mortality risk. Low SVI and new conduction disturbances associated with PPI after THV implantation had negative impact on mid-term outcome in post-TAVI HF-patients.

## 1. Introduction

The indication for transcatheter aortic valve implantation (TAVI) in normal-flow normal-gradient (NFNG) aortic valve stenosis (AS) has developed towards low-risk spectrum and low-symptomatic spectrum [1,2,3] in recent years, accompanied by the progressive improvement and the excellent results. In contrast, in the field of HF patients with low-flow–low-gradient (LFLG) AS knowledge is scarce. In particular, the correct selection of patients for TAVI is difficult. Evidence for the effectiveness of TAVI in the LFLG subpopulation—especially during longer follow-up—has still to be proven. The GWTG score is calculated as previously described [4,5] using seven predictor variables (race, age, systolic blood pressure, heart rate, serum urea nitrogen, sodium and chronic obstructive pulmonary disease). The score is a known predictor of mortality in patients with chronic heart failure (HF). Therefore, we investigated whether this score can be used to predict mortality after transcatheter heart valve (THV) in the subgroup of LFLG-AS.

## 2. Methods

### 2.1. Patient Population

A total of 212 consecutive HF patients with severe LFLG-AS underwent TAVI between January 2017–May 2021 at St. Johannes Hospital Dortmund. These 212 THV-treated patients were divided into a low-risk (*n* = 128) and a high-risk group (*n* = 84) according to the get-with-the-guidelines heart-failure risk score (GWTG-HF). All patients underwent the same diagnostic algorithm including echocardiography, multislice cardiac computed tomography and invasive cardiac catheterization to obtain the necessary parameters. An interdisciplinary heart team discussed all cases beforehand and reached a consensus on the therapeutic strategy. Written informed consent was provided by all patients.

### 2.2. Definition of Low-Flow–Low-Gradient Aortic Valve Stenosis

Severe LFLG-AS was defined according to the current guidelines [6,7]. Severe low-gradient AS was defined as a valve opening (AVA) <1.0 cm^2^ or indexed <0.6 cm^2^/m^2^ with a mean gradient <40 mmHg and a left ventricular ejection fraction (LVEF) <45%. Low flow was scored on the basis of an indexed stroke volume (SVi) <35 mL/m^2^. To delineate paradoxical LFLG-AS, regular computed tomography with quantification of aortic valve calcification and, in individual cases, stress echocardiography was performed [6,8].

### 2.3. Definition of Endpoints and Follow-Up

Clinical and procedural endpoints were categorized according to the updated Valve Academic Research Consortium (VARC-2) criteria [9]. Clinical endpoints were all-cause mortality and readmission for heart failure (CHF) at 1 year. In addition, an analysis of the composite endpoint of all-cause mortality and readmission for heart failure was performed. Analysis of intrahospital events and echocardiographic examination before discharge was also performed. Further follow-up was conducted by outpatient or inpatient visits to the heart center, by contact with the treating physicians or by telephone contact with the patient.

### 2.4. Statistical Analysis

Continuous variables were compared with the student t-test or Mann–Whitney U-test described and described as mean with the respective SD or median with the respective interquartile range. To classify the patients according to their risk profile, the GWTG risk score was evaluated for each individual patient and the population was divided into low (≤43; *n* = 108), intermediate (44–53; *n* = 90) and high risk (≥54; *n* = 14). The performance of the score was investigated using area-under-the-curve (AUC) analysis. Event rates at 1 year were calculated for each group using Kaplan–Meier estimates. Event rates with respective hazard ratios including 95% confidence intervals (CIs) were calculated using Cox regression, and differences between groups were calculated using the log-rank test. A two-sided *p*-value < 0.05 was considered statistically significant. The software RStudio version 1.2.5042 (R Foundation for Statistical Computing, Vienna, Austria) was used for all analyses.

## 3. Results

### 3.1. Patient Population

A total of 212 HF patients were enrolled in the study. Median age was 81.0 [77.0; 84.0], and 34.4% were female. Patients were at intermediate to high surgical risk group according to the European System for Cardiac Operative Risk Evaluation (EuroSCORE II) of 6.6 [4.3; 10.7]. Most patients (94.8%) had severe symptoms according to New York Heart Association functional class (NYHA III or IV). Baseline characteristics of the study population according to risk level are shown in Table 1.

### 3.2. Clinical Outcome

Transcatheter treatment was predominantly conducted via transfemoral access (95.3%) with a wide range of available prostheses. A total of 40.0% (85) patients received an ACURATE *neo/neo2* (Boston Scientific, Marlborough, MA, USA), 38.2% (81) received a SAPIEN 3 (Edwards Lifesciences, Irvine, CA, USA), 17.0% (36) received a Portico (Abbott, Chicago, IL, USA), 4.2% (9) received an Evolut (Medtronic, Dublin, Ireland), and 0.5% (1) received a Lotus (Boston Scientific) valve. Primary device success according to the VARC-2 criteria [9] was achieved in 199 (93.9%) of the procedures. Device failure was due to procedural related death in 0.5% (*n* = 1), significant paravalvular leakage (PVL) in 3.3% (*n* = 7), conversion to sternotomy in 1.4% (*n* = 3) and the need for implantation of a second valve (ViV) in 1.4% (*n* = 3), see Table 2. One patient died immediately after conversion to open heart surgery due to device migration. There was no significant difference in terms of serious complications (bleeding, stroke, renal failure, myocardial infarction, new permanent pacemaker (PPI), in-hospital mortality) as shown in Table 3.

### 3.3. Hemodynamic Performance at Discharge

Echocardiography showed a reduction in mean gradient from 26 to 7 mmHg, an improvement in AVA from 0.8 to 1.8 cm^2^, and an improvement in LVEF from 35.0 to 38.5%. Echocardiographic characteristics for each group are shown in Table 1 for baseline and Table 3 for postprocedural outcome. Postprocedural mean and maximum gradients were lower in the high-risk group. There was especially no difference in the rate of moderate and higher PVL (1.9 vs. 4.5 vs. 7.1%, *p* = 0.281).

### 3.4. GWTG HF Score in LFLG-AS Population

**Figure 1:**
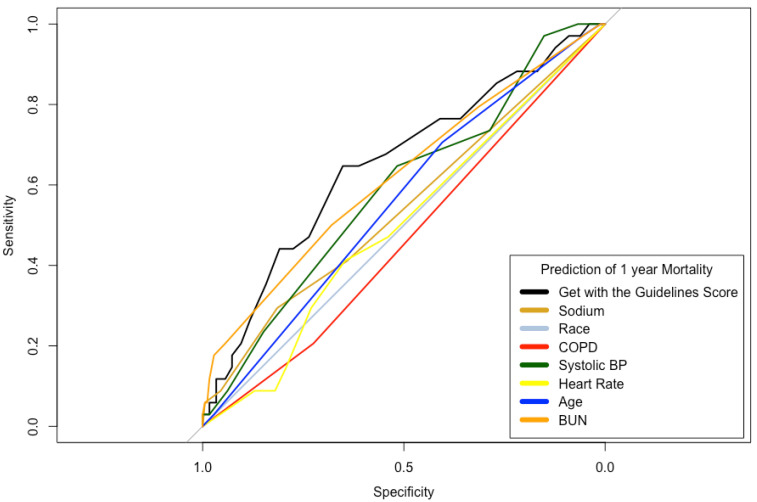
Prediction of mortality. COPD = chronic obstructive pulmonary disease; BP = blood pressure; BUN = blood urea nitrogen. ROC analysis for prediction of mortality (AUC 0.65) with GWTG risk score and its predictor variables.

**Figure 2:**
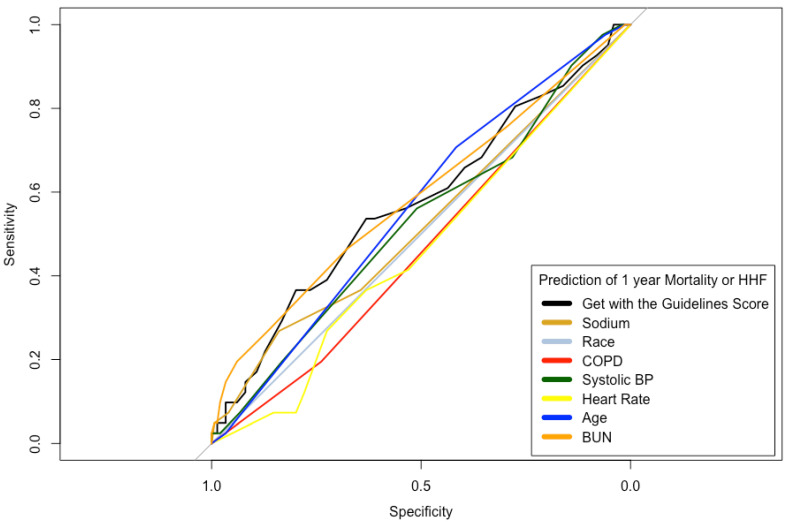
Prediction of composite endpoint (mortality + HHF). ROC analysis for prediction of composite endpoint of mortality and hospitalization for heart failure (AUC 0.57) with GWTG risk score and its predictor variables.

The Euroscore II was increasing with GWTG-HF score level (5.5 vs. 7.2 vs. 9.1, *p* = 0.004). In addition to the parameters integrated in the score, BMI, logistic Euroscore and Euro Score II, creatinine clearance as well as (indexed) stroke volume also differed significantly between groups. When comparing the six (excluding race) integrated parameters of the GWTG-HF score, only sodium showed no significant difference between groups (Table 3). The ROC analysis of the GWTG-HF score and its individual parameters for predicting death and HHF are shown in Figure 1 and Figure 2. In this analysis in relation to 1-year mortality and the combined end point of HHF or mortality, the GWTG-HF score showed an AUC of 0.65 and 0.57 (see Figure 1 and Figure 2). Univariate regression analysis revealed a 1.09-fold (95% CI 1.03–1.15, *p* = 0.004) increased rate of mortality, and a 1.05-fold (95% CI 0.99–1.10, *p* = 0.094) increased incidence of the composite endpoint of HHF and mortality (per one-point increase in GWTG-HF score).

### 3.5. Outcome at 1 Year Follow-Up

**Figure 3:**
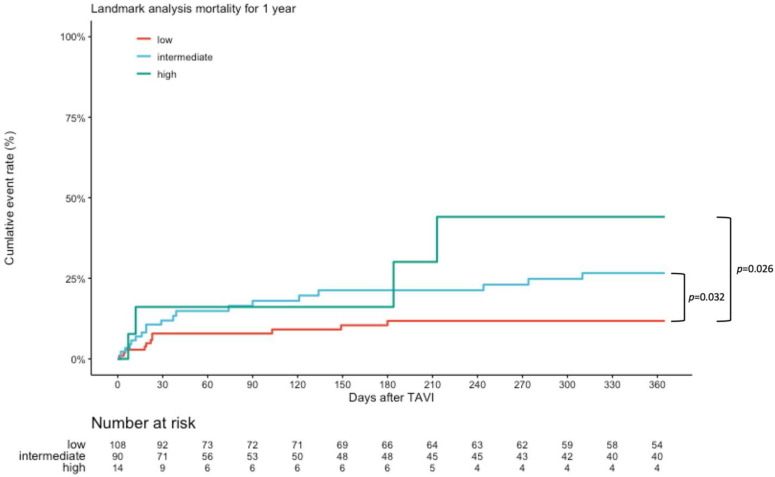
Kaplan–Meier survival curves for all-cause mortality. Kaplan–Meier curves for all-cause mortality during 1-year follow-up. Values are Kaplan Estimates %. HR = Hazard Ratio. Data reported as Kaplan–Meier estimates and competitive risk estimates at the specific time point and do not equal the number of patients with events divided by the total number of patients in each treatment group. The *p* values for event rates were calculated using Log-Rank Test.

**Figure 4:**
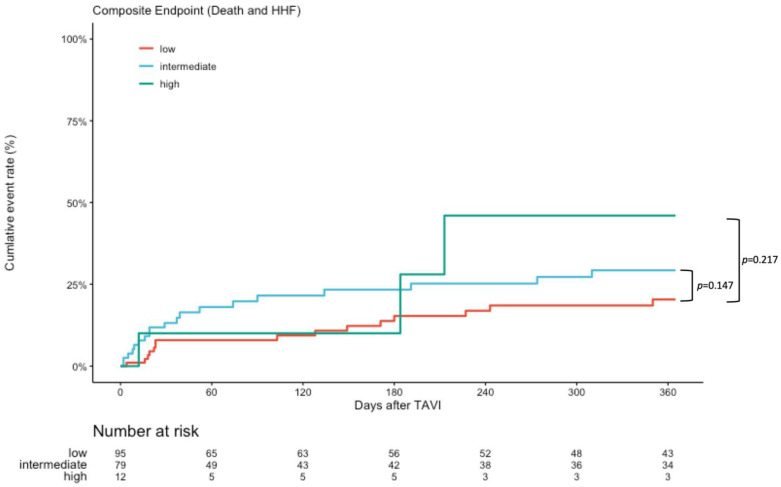
Kaplan–Meier survival curves for composite of HHF and mortality. Kaplan–Meier curves for composite of all-cause mortality and hospitalization for heart failure during 1-year follow-up. Values are Kaplan Estimates %. HR = Hazard Ratio. Data reported as Kaplan–Meier estimates and competitive risk estimates at the specific time point and do not equal the number of patients with events divided by the total number of patients in each treatment group. The *p* values for event rates were calculated using Log-Rank Test.

At 1-year follow-up, 34 (19.1%) of patients had died and 12 (6.9%) were hospitalized due to HF. Kaplan–Meier analysis showed lower event rate in the low-risk subgroup with respect to 1-year all-cause mortality (17.2 vs. 32.2 vs. 50%, *p* = 0.032 and *p* = 0.026), see Figure 3. Composite endpoint of mortality and HHF did not significantly differ (26.3 vs. 35.8 vs. 50%, *p* = 0.147 and *p* = 0.230), see Figure 4. We performed multivariate analysis to evaluate the prediction of mortality by the GWTG-HF score in comparison with known predictors such as LVEF, SVI, new PPI, PVL, and mitral regurgitation. The GWTG score is an independent predictor of mortality (HR 1.07, *p* = 0.030) alongside the rate of associated PPI (HR 3.97, *p* = 0.013). An increase in SVI was protective (HR 0.90; *p* = 0.006), see Table 4.

## 4. Discussion

Risk stratification of HF patients with LFLG-AS remains difficult. Recognized scores such as the logistic EuroSCORE and STS-Score generally overestimate patient mortality. The collective of patients with LFLG-AS is especially heterogenous in terms of hemodynamics and symptomatology. In the present study, we analyzed the GWTG-HF score as a widely accepted risk calculator for heart failure in terms of predicting 1-year mortality and readmission due to HF in the LFLG-AS collective. Originally designed by the American Heart Association’s GWTG-HF program to calculate mortality risk in acute heart failure, the score has also been used in individual studies to calculate risk for mortality or rehospitalization for heart failure after discharge [10] as in valvular heart disease [11,12]. The LFLG-AS collective, in contrast to the NFNG-AS collective, differs strongly with regard to the rate of comorbidities such as renal failure and especially with regard to hemodynamics. Taking into account the hemodynamic characteristics of the collective, a risk stratification model from the heart failure domain seems more appropriate to estimate the mortality risk in this subset. We showed that the GWTG-HF risk score is suitable to predict mortality in the LFLG-AS collective.

### 4.1. Clinical Outcome

Previous studies have shown good results for transcatheter treatment of LFLG-AS compared with surgical or medical treatment regimens alone [13,14]. The rate of primary device success in our cohort was comparably high (93.9%) for the LFLG-AS population [13]. There was no significant difference in serious complications (hemorrhage, stroke, renal failure, myocardial infarction, new PPI, or inhospital mortality) and no difference in clinical outcome and short follow-up in mortality and hospitalization for HF according to the GWTG-HF score. Intrahospital rate of major stroke (2.8%), new PPI (15.8%), coronary obstruction (0.0%) and in-hospital mortality (*n* = 16; 7.5%) was considerably low [15]. In-hospital mortality was due to cardiac (*n* = 9), septic (*n* = 4), hemorrhagic (*n* = 1) and neurologic (*n* = 2) reasons.

### 4.2. Follow Up at 1 Year

We demonstrated that the GWTG-HF score is able to predict mortality at 1 year in HF patients undergoing TAVI. We were not able to show a significant prediction of hospitalization for heart failure nor the combined endpoint of HHF and mortality at 1 year. In addition, multivariate analysis suggests an independent association of the GWTG-HF score with mortality after adjustment for clinically relevant variables such as LVEF, SVI, associated PPI, PVL, and mitral regurgitation [16,17]. In addition to an increased GWTG-HF score, reduced SVI and PPI also turned out to be possible predictors. The association of PPI after TAVI with reduced long-term survival is discussed controversially. However, it is intuitive that especially in the cohort of LFLG, an altered contractility due to conduction abnormalities may have influence the long-term outcome. Our data support the generally practiced cautiousness in the implantation of pacemaker after TAVI, especially in LFLG-AS. For this purpose, screening for potential predictors is recommended [18]. Consideration of SVI has been shown to be useful measure in previous studies in predicting mortality in addition to LFLG-AS collectively in the evaluation of TAVI patients [17,19]. Considerable studies marked elevated mortality in LFLG-AS versus NFNG-AS [15,20]. The data of the intrahospital and short follow-up prove the efficacy and safety of the transcatheter procedure also in patients with LFLG-AS. However, an influence of heart failure on mortality is only evident in the follow-up after 1 year. Here, in addition to PPI and SVI, heart failure in particular seems to determine the risk of death and hospitalization.

### 4.3. Limitations

This study has the inherent limitation of any observational study with limited sample size considering the specific clinical profile. Especially the low number of patients in the high-risk group is a limitation and may introduce bias into the results. The data correspond to a real-world study and reflect current clinical practice in a high-volume center. Outcome data describe mid-term results; future long-term confirmation after years is essential.

## 5. Conclusions

The outcome of TAVI in subset of LFLG-AS is good. The GWTG-HF score predicts outcome in LFLG-AS HF-patients after TAVI with respect to 1-year mortality. The GWTG-HF score could easily be used to identify high-risk patients requiring more strict and closer long-term follow-up monitoring after intervention. Overall, especially short-term follow-up after TAVI for LFLG-AS showed reassuring results in HF patients. Moreover, SVI and PPI were relevant prognostic markers in the LFLG-AS collective.

## Figures and Tables

**Table 1:** Baseline characteristics.

	Low Risk	Intermediate	High Risk	*p*-Value
	*n* = 108	*n* = 90	*n* = 14	
**Baseline Characteristics**				
Age, years	79.0 [75.0; 82.0]	82.0 [79.2; 84.0]	86.0 [82.2; 88.8]	<0.001
Gender, female	39 (36.1%)	31 (34.4%)	3 (21.4%)	0.612
BMI, kg/m^2^	27.6 [24.3; 30.8]	26.2 [24.0; 29.4]	24.4 [22.9; 27.8]	0.036
Log. Euroscore, %	17.3 [11.6; 25.1]	21.9 [14.8; 31.8]	36.0 [20.9; 48.4]	<0.001
Euroscore II, %	5.5 [3.9; 8.8]	7.2 [4.9; 11.3]	9.1 [6.1; 15.9]	0.004
NYHA class III/IV	103 (95.4%)	86 (95.6%)	12 (85.7%)	0.284
Previous COPD	23 (21.3%)	27 (30.0%)	6 (42.9%)	0.130
Previous aHT	104 (96.3%)	83 (92.2%)	12 (85.7%)	0.125
Syst. RR, mmHg	138 [130; 146]	120 [108; 127]	101 [93; 120]	<0.001
Diabetes	45 (41.7%)	42 (46.7%)	5 (35.7%)	0.651
Previous Dialysis	3 (2.8%)	5 (5.6%)	2 (14.3%)	0.127
Previous PAD	30 (27.8%)	18 (20.0%)	6 (42.9%)	0.140
Previous Stroke	24 (22.2%)	12 (13.3%)	1 (7.1%)	0.186
Previous CAD	80 (74.1%)	67 (74.4%)	10 (71.4%)	0.939
Previous MI	15 (13.9%)	12 (13.3%)	3 (21.4%)	0.666
Previous PCI	55 (50.9%)	38 (42.2%)	7 (50.0%)	0.463
Previous CABG	16 (14.8%)	14 (15.6%)	0 (0.0%)	0.359
**Echocardiographic Characteristics**				
LVEF, %	36.0 [28.8; 40.0]	33.0 [25.2; 39.0]	33.5 [28.2; 40.0]	0.085
AVA, cm^2^	0.8 [0.6; 0.9]	0.7 [0.6; 0.9]	0.7 [0.6; 0.8]	0.050
SV, mL	55.0 [44.0; 64.2]	48.0 [39.2; 55.8]	47.5 [38.8; 51.0]	0.001
SVi, mL/m^2^	28.2 [23.9; 32.1]	25.8 [20.0; 29.0]	25.0 [21.8; 28.2]	0.004
Pmean, mmHg	26.5 [21.0; 32.0]	25.0 [18.0; 31.0]	24.5 [22.2; 31.2]	0.265
Pmax, mmHg	45.0 [36.5; 50.0]	42.0 [31.0; 50.0]	43.0 [38.5; 50.0]	0.471
MR ( ≥ III)	3 (2.8%)	2 (2.2%)	0 (0.0%)	1.000
TR ( ≥ III)	0 (0.0%)	1 (1.1%)	1 (7.1%)	0.060
pAH ^1^	5 (4.6%)	7 (8.0%)	1 (7.1%)	0.515
**Electrocardiographic Characteristics**				
Atrial fibrillation	55 (50.9%)	53 (58.9%)	9 (64.3%)	0.415
Heart rate, bpm	72.0 [64.8; 81.2]	82.0 [70.5; 99.5]	99.5 [88.5; 112.0]	<0.001
LBBB	17 (15.7%)	16 (18.0%)	2 (14.3%)	0.914
RBBB	12 (11.1%)	8 (9.0%)	4 (28.6%)	0.122
New pacemaker	35 (32.4%)	24 (26.7%)	1 (7.1%)	0.131
**Multisliced Computed Tomography Data (MSCT)**				
Area cm^2^	5.0 [4.4; 5.7]	5.3 [4.6; 6.0]	4.8 [4.6; 5.6]	0.262
Diameter min., mm	22.4 [20.5; 24.0]	23.0 [21.3; 24.5]	21.7 [21.0; 23.8]	0.132
Diameter max., mm	28.7 [27.2; 30.9]	29.3 [27.6; 31.2]	29.1 [27.2; 30.9]	0.362
Eccentricity	0.2 [0.2; 0.3]	0.2 [0.2; 0.3]	0.2 [0.2; 0.3]	0.800
Calcification, AU	1927 [1163; 2807]	2089 [1474; 2908]	2316 [1553; 3155]	0.376
**Laboratory Characteristics**				
eGFR, mL/min	58.5 [43.0; 77.0]	46.5 [35.0; 59.0]	33.5 [22.0; 47.0]	<0.001
Sodium, mmol/L	139 [137; 141]	140 [137; 141]	139 [135; 141]	0.405
BUN, mg/dL	21.1 [16.8; 26.1]	29.6 [23.1; 37.5]	44.9 [32.9; 68.1]	<0.001
CRP, mg/dL	5.8 [1.8; 12.7]	9.4 [4.0; 17.3]	9.5 [6.0; 30.2]	0.027
Haemoglobin, g/dL	12.7 [11.7; 13.8]	12.2 [11.3; 13.3]	11.4 [10.1; 13.0]	0.043

**Abbreviations:** Values are mean SD, *n* (%), or median (interquartile range). BMI = body mass index; aHT = arterial hypertension; RR = arterial pressure; CAD = coronary artery disease; PAD = peripheral artery disease; MI = myocardial infarction; CABG = coronary artery bypass grafting; COPD = chronic obstructive pulmonary disease; LVEF = left ventricular ejection fraction; AVA = aortic valve area; Pmean = mean transaortic gradient; Pmax = maximum transaortic gradient; SV = stroke volume; SVi = indexed stroke volume; TR = Tricuspid regurgitation; MR = Mitral regurgitation; AF = Atrial fibrillation; MSCT = multisliced computed tomography; NYHA = New York Heart Association functional class; PCI = percutaneous coronary intervention; LBBB = complete left bundle branch block; RBBB = complete right bundle branch block; ^1^ pAH = systolic pulmonary artery pressure (sPAP) > 60 mmHg; AU = Agatston Units; eGFR = estimated glomerular filtration rate; CRP = C-reactive protein; BUN = blood urea nitrogen.

**Table 2:** Device failure.

	Low Risk	Intermediate Risk	High Risk	*p*-Value
	*n* = 108	*n* = 90	*n* = 14	
**Device Failure ^1^**	6 (5.6%)	6 (6.8%)	1 (7.7%)	0.731
Procedural-related death *	1 (0.5%)	0 (0.0%)	0 (0.0%)	1.000
Intended performance	106 (99.1%)	86 (95.6%)	14 (100.0%)	0.325
Significant PVL ^2^	2 (1.9%)	4 (4.5%)	1 (7.1%)	0.281
Elevated gradient ^3^	2 (1.9%)	1 (1.1%)	0 (0.0%)	1.000
Multiple valves	1 (0.9%)	2 (2.2%)	0 (0.0%)	0.669
Conversion to sternotomy *	1 (0.9%)	2 (2.2%)	0 (0.0%)	0.668

**Abbrevations:** Values are mean SD, *n* (%). PVL = paravalvular leakage. ^1^ Prothesis mismatch, mean aortic gradient ≥ 20 mmHg or peak velocity ≥ 3 m/s, moderate or severe prosthetic valve aortic regurgitation of the first implanted valve, multiple events possible. ^2^ Significant PVL ≥ Grade II. ^3^ Elevated gradient ≥ 20 mmHg. * One patient died after conversion to sternotomy due to device migration.

**Table 3:** Periprocedural findings.

	Low Risk	Intermediate	High Risk	*p*-Value
	*n* = 108	*n* = 90	*n* = 14	
**Procedural Data**				
Transfemoral Access	100 (92.6%)	89 (98.9%)	13 (92.9%)	0.064
Pre dilatation	55 (50.9%)	50 (56.2%)	7 (50.0%)	0.742
Post dilatation	20 (18.7%)	9 (10.2%)	3 (21.4%)	0.162
Procedural time, min	57 [45; 75]	56 [45; 65]	59 [50; 65]	0.571
Contrast, mL	110 [90; 140]	106 [90; 130]	110 [100; 130]	0.434
Conscious sedation	97 (89.8%)	80 (88.9%)	11 (78.6%)	0.425
**Postprocedural echocardiographic outcome**				
AVA, cm^2^	1.9 [1.6; 2.1]	1.7 [1.5; 2.2]	1.9 [1.8; 2.2]	0.518
LVEF	40 [31; 45]	36 [30; 45]	38 [28; 49]	0.620
Pmean, mmHg	7.0 [5.0; 9.0]	7.0 [5.0; 9.0]	5.0 [4.0; 6.0]	0.011
Pmax, mmHg	13.0 [10.0; 17.8]	13.0 [9.0; 18.0]	10.0 [9.0; 10.0]	0.013
Significant PVL ^1^	2 (1.9%)	4 (4.5%)	1 (7.1%)	0.281
**Adverse events**				
Major Stroke	2 (1.9%)	4 (4.4%)	0 (0.0%)	0.613
Major vascular complications	6 (5.6%)	3 (3.3%)	0 (0.0%)	0.741
Life threatening bleeding	4 (3.7%)	1 (1.1%)	0 (0.0%)	0.560
Renal failure (≥2)	5 (5.1%)	5 (6.0%)	2 (14.3%)	0.343
Coronary artery obstruction	0 (0.0%)	0 (0.0%)	0 (0.0%)	1.000
New permanent pacemaker ^2^	12 (16.44%)	9 (13.64%)	3 (23.08%)	0.631
**In hospital**				
Days in hospital	7.0 [5.0; 9.0]	7.0 [5.0; 10.0]	7.0 [6.0; 8.8]	0.288
Days on intensive care unit	2.0 [1.0; 3.0]	2.0 [1.0; 3.0]	3.0 [2.0; 3.0]	0.094
In-hospital mortality	7 (6.5%)	7 (7.8%)	2 (14.3%)	0.390

**Abbreviations:** Values are mean SD, *n* (%), or median (interquartile range). ^1^ Significant PVL ≥ Grade II. ^2^ Excluded patients with pacemaker at baseline (*n* = 60). AVA = aortic valve area; LVEF = left ventricular ejection fraction; PVL = paravalvular leakage. Pmean = mean transaortic gradient. Pmax = maximum transaortic gradient.

**Table 4:** Multivariate logistic regression (predictors for mortality after 1 year).

Predictor	HR (CI 95%)	*p*-Value
GWTG-HF Score	1.07 (1.01–1.14)	0.030
Significant PVL (≥Grade II)	2.44 (0.37–16.25)	0.357
Mitral Regurgitation (≥Grade III)	1.93 (0.24–15.32)	0.532
Left ventricular ejection fraction (per 1% increase)	1.02 (0.97–1.08)	0.352
Stroke volume index (per 1 mL/m^2^ increase)	0.90 (0.83–0.97)	0.006
Post mean transaortic gradient (per 1 mmHg increase)	0.96 (0.86–1.07)	0.496
New pacemaker implantation	3.97 (1.34–11.75)	0.013

**Abbreviation:** PVL = paravalvular regurgitation.

## Data Availability

Data is contained within the article.

## Abbreviations

AUC	area under the curve
GWTG-HF	get-with-the-guidelines heart-failure
HF	heart failure
HHF	hospitalization for heart failure
LFLG	low-flow–low-gradient
PPI	permanent pacemaker implantation
PVL	paravalvular regurgitation
ROC	receiver operating characteristics
TAVI	transcatheter aortic valve implantation
THV	transcatheter heart valve
